# Conjugation Magnetic PAEEP-PLLA Nanoparticles with Lactoferrin as a Specific Targeting MRI Contrast Agent for Detection of Brain Glioma in Rats

**DOI:** 10.1186/s11671-016-1421-x

**Published:** 2016-04-27

**Authors:** Binhua Luo, Siqi Wang, Rong Rao, Xuhan Liu, Haibo Xu, Yun Wu, Xiangliang Yang, Wei Liu

**Affiliations:** College of Life Science and Technology, Huazhong University of Science and Technology, 1037 Luoyu Road, Wuhan, 430074 China; College of Pharmacy, Hubei University of Science and Technology, Xianning, Hubei China; Department of Radiology, Union Hospital, Tongji Medical College, Huazhong University of Science and Technology, 1277 Jiefang Avenue, Wuhan, 430022 People’s Republic of China; National Engineering Research Center for Nanomedicine, Huazhong University of Science and Technology, Wuhan, China; Department of Radiology, Zhongnan Hospital of Wuhan University, Wuhan, 430071 China; Department of Biomedical Engineering, University at Buffalo, State University of New York, Buffalo, USA

**Keywords:** Poly(aminoethyl ethylene phosphate)/poly (L-lactide) copolymer, Oleylamine-coated Fe_3_O_4_ nanoparticles, Lactoferrin, Superparamagnetic, MRI contrast agent, Brain glioma targeting

## Abstract

The diagnosis of malignant brain gliomas is largely based on magnetic resonance imaging (MRI) with contrast agents. In recent years, nano-sized contrast agents have been developed for improved MRI diagnosis. In this study, oleylamine-coated Fe_3_O_4_ magnetic nanoparticles (OAM-MNPs) were synthesized with thermal decomposition method and encapsulated in novel amphiphilic poly(aminoethyl ethylene phosphate)/poly(L-lactide) (PAEEP-PLLA) copolymer nanoparticles. The OAM-MNP-loaded PAEEP-PLLA nanoparticles (M-PAEEP-PLLA-NPs) were further conjugated with lactoferrin (Lf) for glioma tumor targeting. The Lf-conjugated M-PAEEP-PLLA-NPs (Lf-M-PAEEP-PLLA-NPs) were characterized by photon correlation spectroscopy (PCS), transmission electron microscopy (TEM), Fourier transform infrared (FTIR), thermo-gravimetric analysis (TGA), X-ray diffraction (XRD), and vibrating sample magnetometer (VSM). The average size of OAM-MNPs, M-PAEEP-PLLA-NPs, and Lf-M-PAEEP-PLLA-NPs were 8.6 ± 0.3, 165.7 ± 0.6, and 218.2 ± 0.4 nm, with polydispersity index (PDI) of 0.185 ± 0.023, 0.192 ± 0.021, and 0.224 ± 0.036, respectively. TEM imaging showed that OAM-MNPs were monodisperse and encapsulated in Lf-M-PAEEP-PLLA-NPs. TGA analysis showed that the content of iron oxide nanoparticles was 92.8 % in OAM-MNPs and 45.2 % in Lf-M-PAEEP-PLLA-NPs. VSM results indicated that both OAM-MNPs and Lf-M-PAEEP-PLLA-NPs were superparamagnetic, and the saturated magnetic intensity were 77.1 and 74.8 emu/g Fe. Lf-M-PAEEP-PLLA-NPs exhibited good biocompatibility in cytotoxicity assay. The high cellular uptake of Lf-M-PAEEP-PLLA-NPs in C6 cells indicated that Lf provided effective targeting for the brain tumor cells. The *T*_2_ relaxation rate (*r*_2_) of M-PAEEP-PLLA-NPs and Lf-M-PAEEP-PLLA-NPs were calculated to be 167.2 and 151.3 mM^−1^ s^−1^. In MRI on Wistar rat-bearing glioma tumor, significant contrast enhancement could clearly appear at 4 h after injection and last 48 h. Prussian blue staining of the section clearly showed the retention of Lf-M-PAEEP-PLLA-NPs in tumor tissues. The results from the in vitro and in vivo MRI indicated that Lf-M-PAEEP-PLLA-NPs possessed strong, long-lasting, tumor targeting, and enhanced tumor MRI contrast ability. Lf-M-PAEEP-PLLA-NPs represent a promising nano-sized MRI contrast agent for brain glioma targeting MRI.

## Background

Malignant glioma is a primary brain tumor that originates from the supportive cells of the brain called glial cells. Because of the high proliferation of blood vessels and endothelial cells, a major pathophysiological feature of gliomas is their ability to diffuse and invade into surrounding brain tissue [1]. Although there are significant advances in neuroimaging and neurosurgical techniques over the past 30 years, the median survival time of glioblastoma patients is only 12 to 18 months after surgery [2, 3].

The diagnosis of malignant gliomas is largely based on radiographic findings in the setting of a typical clinical presentation and biopsy specimen obtained invasively [4]. Magnetic resonance imaging (MRI), a much easier, safer, and non-invasive tool, has been widely used in clinical diagnosis [5]. MRI is very effective in characterizing soft tissue because of its high tissue resolution [6]. Current approach for diagnosis and grading of brain glioma is MRI with administration of gadolinium (Gd) chelate-based contrast agents [7]. However, such agents have several shortcomings, such as short imaging time, fast diffusion, and side effect of nephrogenic systemic fibrosis. And Gd-enhanced *T*_1_-weighted MRI images could not distinguish effectively various etiologies, such as tumor regrowth and radiation necrosis [8].

In recent years, superparamagnetic iron oxide nanoparticles (SPIONs) have been developed as MRI contrast agents for diagnosis of malignant gliomas [9]. SPIONs are usually less than 10 nm, which are useful as *T*_2_ contrast agents owning to more effective at shortening *T*_2_ rather than *T*_1_ relaxation time. SPIONs can generate contrast signals at higher level than paramagnetic Gd chelates, providing an opportunity for the early detection of tumors, and SPIONs display outstanding biocompatibility and biodegradability [10]. Current approaches to developing SPION-based MRI contrast agents with long-time stability and specificity to target tumor are to incorporate iron oxide into polymeric nanocarriers [10, 11]. The polymeric nanocarriers with incorporation of iron oxide revealed higher relaxivity than dissociated monocrystalline iron oxide particles. The encapsulation of iron oxide could shield them from interacting with vascular components, as well as the sustain release of free iron ions, which may ultimately reduce the side effects [12]. Furthermore, the polymeric nanocarriers can conjugate tumor-targeting molecules for providing brain glioma-specific detection. However, the cytotoxicity, non-biodegradable, and limitation in functional possibility and flexibility restrict the clinical application of polymers as MRI contrast agent [13, 14]. Moreover, previous studies have focused on enhancing the contrast capabilities of the polymeric nanocarriers with incorporation of iron oxide, and their active tumor-targeting potential has not been well explored.

We have successfully synthesized a novel amphiphilic poly(aminoethyl ethylene phosphate)/poly(L-lactide) (PAEEP-PLLA) copolymer by ring-opening polymerization reaction, which contains hydrophobic PLLA and hydrophilic PAEEP segments with good biocompatibility and biodegradability [15]. The PAEEP segment containing a large number of amino groups can be modified with tumor-targeting molecules, such as lactoferrin (Lf), an effective targeting ligand for gliomas. In this study, oleylamine-coated Fe_3_O_4_ magnetic nanoparticles (OAM-MNPs) were synthesized and encapsulated in PAEEP-PLLA nanoparticles (M-PAEEP-PLLA-NPs). M-PAEEP-PLLA-NPs were further conjugated with Lf (Lf-M-PAEEP-PLLA-NPs) for glioma tumor targeting. The physiochemical properties, biocompatibility, and the brain glioma targeting MRI effect of Lf-M-PAEEP-PLLA-NPs in vitro and in vivo were studied.

## Methods

### Materials

Iron (III) acetylacetonate (Fe(acac)_3_), oleylamine, and benzyl ether were purchased from Acros Organics (Geel, Belgium). Lf from bovine colostrum and fluorescent aminoluciferin were purchased from Sigma-Aldrich (St. Louis, MO, USA). Poly(vinyl alcohol) (PVA-217, 1700 of polymerization degree, 88.5 % of hydrolysis degree) was obtained from Kuraray (Tokyo, Japan). Chloroform was purchased from Sinopharm (Shanghai, China). α-Malemidyl-ω-*N*-hydroxysuccinimidylpoly(ethylene glycol) (NHS-PEG-MAL, MW3400) was obtained from Jenkem Technology (Beijing, China). Male Wistar rats (250–300 g) were supplied by Hubei Center for Disease Control and Prevention (Animal Qualification Certificate No. 4200060003926). All other reagents were of analytical grade.

### Synthesis of OAM-MNPs

OAM-MNPs were synthesized using the thermal decomposition method as described previously [16]. Briefly, 3 mmol Fe(acac)_3_ was dispersed in a solution of 7.5 mL oleylamine and 7.5 mL benzyl ether. The mixture was dehydrated at 110 °C for 1 h under nitrogen atmosphere with magnetic stirring, then heated to 298 °C at a rate of 15 °C/5min, and aged at 298 °C for 1 h. The solution was cooled to room temperature, added with ethanol for precipitation and centrifuged at 7000 rpm for 10 min. The precipitates containing OAM-MNPs were harvested, washed with ethanol three times, and dried at 60 °C overnight.

### Preparation of Lf-M-PAEEP-PLLA-NPs

M-PAEEP-PLLA-NPs were prepared by ultrasonic emulsification and solvent evaporation method. PAEEP-PLLA (100 mg) and OAM-MNPs (100 mg) were dissolved in 1 mL chloroform. The mixture was drop-wise added into 10 mL 0.3 % PVA solution with 2-min sonication at 100 W. The formed oil/water (O/W) emulsion was stirred at room temperature overnight and centrifuged at 7000 rpm for 10 min. The precipitates were resuspended in normal saline solution and stored at 4 °C.

The conjugation of M-PAEEP-PLLA-NPs with Lf was performed through a PEG spacer as described in our previous work [15]. First, sulfhydryl groups were linked to Lf molecules using Traut’s reagent; the product obtained was Lf sulfhydryl. Second, the M-PAEEP-PLLA-NPs was incubated with α-malemidyl-ω-*N*-hydroxysuccinimidylpoly(ethylene glycol). Finally, glycine solution was added to quench the reaction and the obtained mixture was incubated with Lf sulfhydryl. The Lf-M-PAEEP-PLLA-NPs were purified by centrifugation. The formation of OAM-MNPs, M-PAEEP-PLLA-NPs, and Lf-M-PAEEP-PLLA-NPs were monitored by Fourier transform infrared spectroscopy (FTIR, VERTEX 70, Germany).

### Characterization of OAM-MNPs and Lf-M-PAEEP-PLLA-NPs

The surface morphology of OAM-MNPs and Lf-M-PAEEP-PLLA-NPs were examined using transmission electron microscopy (TEM, JEM-2010, Japan) at an accelerating voltage of 100 kV. The hydrodynamic size and polydispersity index (PDI) were measured by photon correlation spectroscopy (PCS, Nano-ZS90 zetasizer, UK) at 25 °C using He-Ne laser of 633 nm.

Thermogravimetric analyses (TGA) of OAM-MNPs and Lf-M-PAEEP-PLLA-NPs were obtained with a thermogravimetric analyzer (EXSTAR TG/DTA 6000 Series, Japan); 10 mg sample was heated at 100 °C for 30 min to remove all free water. The pyrolysis property was examined from 100 to 960° C heated at a constant rate of 15 °C/min. Nitrogen was used as the purge gas at a 50-mL/min flow rate for the thermal stability.

The crystal structures of OAM-MNPs and Lf-M-PAEEP-PLLA-NPs were studied by X-ray diffraction (XRD) on a diffractometer (X′Pert Powder, Netherlands) with Cu Kα radiation(40 kV, 100 mA, and *λ* = 0.1541 nm). Patterns were recorded in the continuous scanning mode from 10 to 90 °C with a step size of 0.0131° and a step time of 9.945 s.

The magnetic properties of OAM-MNPs and Lf-M-PAEEP-PLLA-NPs were characterized by the vibrating sample magnetometer (VSM, Model 7404, USA) using maximum fields of 15,000 Oe at room temperature.

### Cytotoxicity Assay

Rat C6 glioma (C6 cells) and human normal liver cell line (HL-7702) were purchased from Shanghai Institute of Life Science Cell Culture Center (Shanghai, China). C6 cells and HL-7702 cells were cultured in Dulbecco’s modified Eagle’s medium (DMEM) with 10 % fetal calf serum (FCS) (Gibco, Thermo Fisher Scientific, Waltham, MA, USA), penicillin (100 IU/mL), and streptomycin (100 mg/mL), in humidified air containing 5 % CO_2_ at 37 °C. The in vitro cytotoxicity was evaluated by MTT assay. Cells were seeded at 5 × 10^3^ cells/well in 96-well plates and incubated overnight; 0.1 mL of DMEM (control), M-PAEEP-PLLA-NPs, or Lf-M-PAEEP-PLLA-NPs solution (12.5, 25, 50, 75, 100 μg/mL) was added and incubated for 24 or 48 h. Then, 20 μL of MTT solution (5 mg/mL) was added to each well and the plate was incubated at 37 °C for 4 h. After incubation, the absorbance was measured at 492 nm using a microplate reader (318C-Microplate Reader, China).

### Effect of Lf on Cellular Uptake

C6 cells and ECV304 cells were used to investigate the cellular uptake of Lf-M-PAEEP-PLLA-NPs and M-PAEEP-PLLA-NPs. Cells were seeded in 12-well plates at a density of 150,000 cells/well in DMEM and incubated for 24 h. Then, Lf-M-PAEEP-PLLA-NPs or M-PAEEP-PLLA-NPs was added in cell culture medium at concentrations of 5, 10, 15, and 20 μg/mL. After 2-h incubation, the medium was removed and cells were washed with PBS for three times. To remove cell debris, samples were centrifuged at 2000 rpm for 10 min, followed by resuspending in double distilled water. The supernatant was analyzed by flow cytometry (Beckman Coulter, France). Fluorescent aminoluciferin was labeled onto the nanoparticles for the flow cytometric cell uptake study.

### In Vitro MR Imaging

The MR *T*_2_ relaxivity of the nanoparticles was determined using 3.0-T whole-body MR scanner (MAGNETOM Trio, A Tim System 3T, Siemens, Germany) in combination with an eight-channel wrist joint coil. Lf-M-PAEEP-PLLA-NPs and M-PAEEP-PLLA-NPs were diluted in 1 % agarose at an iron concentration range of 0–5 μg/mL and then transferred to a 96-well plate. *T*_2_-weighted images were acquired using a spin echo sequence. The parameters are field of view = 120 mm, base resolution = 384 × 384, slice thickness = 1.5 mm, multiple echo times = 20, 40, 60, 80, 100, 120, and 140 ms, repetition time = 2000 ms, and scan time = 13 min. *T*_2_ relaxation rates were plotted against iron concentrations in the particle dilutions. The relaxivity was determined by a linear fit.

### In Vivo MR Imaging

All animal studies were approved by the Animal Experimentation Ethics Committee of Huazhong University of Science and Technology and carried out in compliance with guidelines approved by the Science and Technology Department of Hubei Province.

Nine rats were anesthetized with 10 % chloral hydrate (i.p., 4 mL/kg) and placed in a stereotactic frame. A burrhole was drilled through the skull 1 mm posterior and 3 mm lateral to the bregma. A 25-μL microinjector was used to inject 10-μL (1 × 10^6^) C6 cells. The injection was done slowly over 10 min and stayed 10 min, and the needle was withdrawn after another 10 min. The skin was closed with nonmagnetic sutures. MRI was performed after tumor growth for 2 weeks as described in previous reports [17–19].

Before MRI examination, all rats were anesthetized with chloral hydrate. Images were obtained before and after injection of contrast agents (2, 4, 8, 12, 24, and 48 h) using a 3.0-T whole-body MRI scanner. The rats were injected with Gd-based contrast agent (Omniscan™, GE Healthcare, USA) to determine the success of the rat model of brain glioma. Then, 1.5 mL M-PAEEP-PLLA-NPs or Lf-M-PAEEP-PLLA-NPs (12 mg Fe/kg) was injected via tail vein, respectively. An eight-channel wrist joint coil was used for MR imaging after the rats were placed in the instrument. The parameters are as follows: TR/TE = 20 ms/8.53 ms, field of view (FOV) = 50 mm, flip angel (FA) = 50°, base resolution = 256 × 256, 80 layer/block, and slice thickness = 2 mm (before and after Gd-DTPA enhanced 3D *T*_1_ weighted imaging); TR/TE = 6540 ms/91 ms, FOV = 35 mm, base resolution = 192 × 192, slice thickness = 1mm, average: 9 (2D*T*_2_WI TSE sequence); TR/TE = 7 ms/16 ms, FOV = 60 mm, slice thickness = 1 mm, FA = 20°, slices per slab = 64, average: 3, voxel size = 0.2 × 0.2 × 1.0 mm, bandwidth = 130 Hz/Px, base resolution = 320 × 320 (3D susceptibility-weighted imaging (SWI)). The signal intensity (SI) of the tumors was determined using a region of interest (ROI) within the defined tumor area and normalized to the SI of the back muscle adjacent to the tumor. Relative signal enhancement (RSE) of tumor was calculated using the following equation: [18]

$$ \mathrm{R}\mathrm{S}\mathrm{E}\left(\%\right)=\left[1-\left({\mathrm{SI}}_{\mathrm{post}\hbox{-} \mathrm{tumor}}/{\mathrm{SI}}_{\mathrm{post}\hbox{-} \mathrm{muscle}}\right)/\left({\mathrm{SI}}_{\mathrm{pre}\hbox{-} \mathrm{tumor}}/{\mathrm{SI}}_{\mathrm{pre}\hbox{-} \mathrm{musle}}\right)\right]\times 100 $$

SI_pre_ was measured before injection and SI_post_ was measured at each follow-up time after injection (2, 4, 6, 8, 12, 24, and 48 h).

### Histopathological Analysis

After MRI, all rats were anaesthetized and perfused with PBS solution followed by 4 % paraformaldehyde. Brain glioma were removed and fixed in 4 % paraformaldehyde for 24 h, embedded in paraffin, slided at 5 μm, and stained with hematoxylin and eosin (H&E) and Prussian blue.

### Statistical Analysis

All data were expressed as mean ± SD. Unpaired student’s *t* test was used to examine the differences among various groups. *P* values less than 0.05 were considered statistically significant. All statistical analyses were performed using SPSS, Version 19.

## Results and Discussion

### Preparation and Characterization of Lf-M-PAEEP-PLLA-NPs

**Fig. 1:**
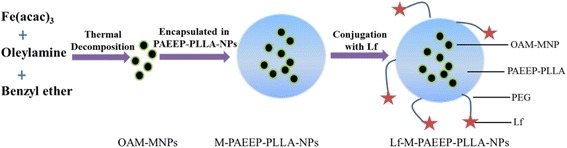
Steps for preparation of Lf-M-PAEEP-PLLA-NPs

Figure 1 showed the workflow of the preparation of Lf-M-PAEEP-PLLA-NPs. Firstly, OAM-MNPs were synthesized using the thermal decomposition method of iron acetylacetonate in oleylamine and benzyl ether. Then, OAM-MNPs were encapsulated in amphiphilic PAEEP-PLLA copolymer nanoparticles by ultrasonic emulsification and solvent evaporation method. Finally, M-PAEEP-PLLA-NPs were conjugated with Lf through a PEG spacer to prepare Lf-M-PAEEP-PLLA-NPs.

In this study, OAM-MNPs were encapsulated in PAEEP-PLLA copolymer nanoparticles. PAEEP segment is highly hydrophilic and PLLA segment is hydrophobic, and both PAEEP and PLLA are biodegradable [20]. So the obtained PAEEP-PLLA block copolymer is amphipathic and biodegradable with the combined appealing features of PAEEP and PLLA. Hydrophilic polyphosphate chains can effectively prevent the nanoparticles from being cleared by RES in the blood. Moreover, PAEEP segment contains a large number of amino groups, which can be readily modified with tumor-targeting molecules. Lf, a mammalian cationic iron-binding glycoprotein belonging to the transferrin family, contains a polypeptide chain with 680–690 amino acids. In our recent work, Lf has been proved as an effective targeting ligand for brain gliomas [17, 18].

**Fig. 2:**
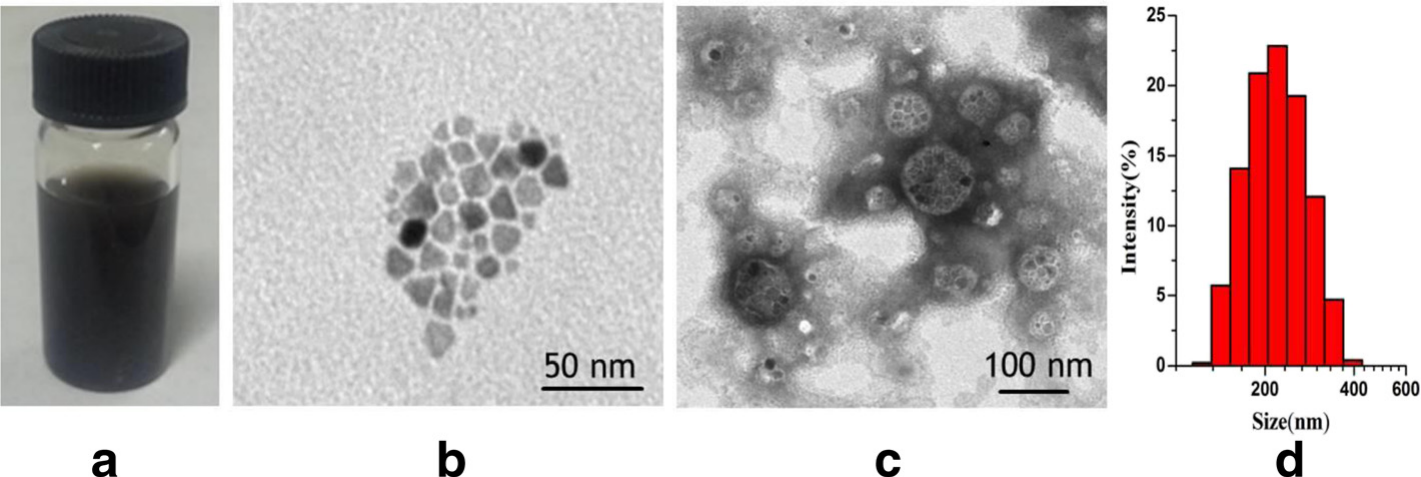
Characterization of Lf-M-PAEEP-PLLA-NPs. **a** Lf-M-PAEEP-PLLA-NPs solution. **b** TEM image of OAM-MNPs. **c** TEM image of Lf-M-PAEEP-PLLA-NPs. **d** Size of Lf-M-PAEEP-PLLA-NPs

The appearance of Lf-M-PAEEP-PLLA-NPs suspension is shown in Fig. 2a. Figure 2b, c shows the TEM images of OAM-MNPs and Lf-M-PAEEP-PLLA-NPs, indicating that hydrophobic OAM-MNPs were monodisperse and successfully encapsulated into Lf-M-PAEEP-PLLA-NPs. PCS measurements showed that the average sizes of OAM-MNPs, M-PAEEP-PLLA-NPs, and Lf-M-PAEEP-PLLA-NPs were 8.6 ± 0.3, 165.7 ± 0.6, and 218.2 ± 0.4 nm, with PDI of 0.185 ± 0.023, 0.192 ± 0.021, and 0.224 ± 0.036, respectively. The zeta potential of Lf-M-PAEEP-PLLA-NPs was −10.4 ± 0.2 mV. The concentration of the conjugated Lf was 0.289 mg/mL, as determined by the bicinchoninic acid method.

**Fig. 3:**
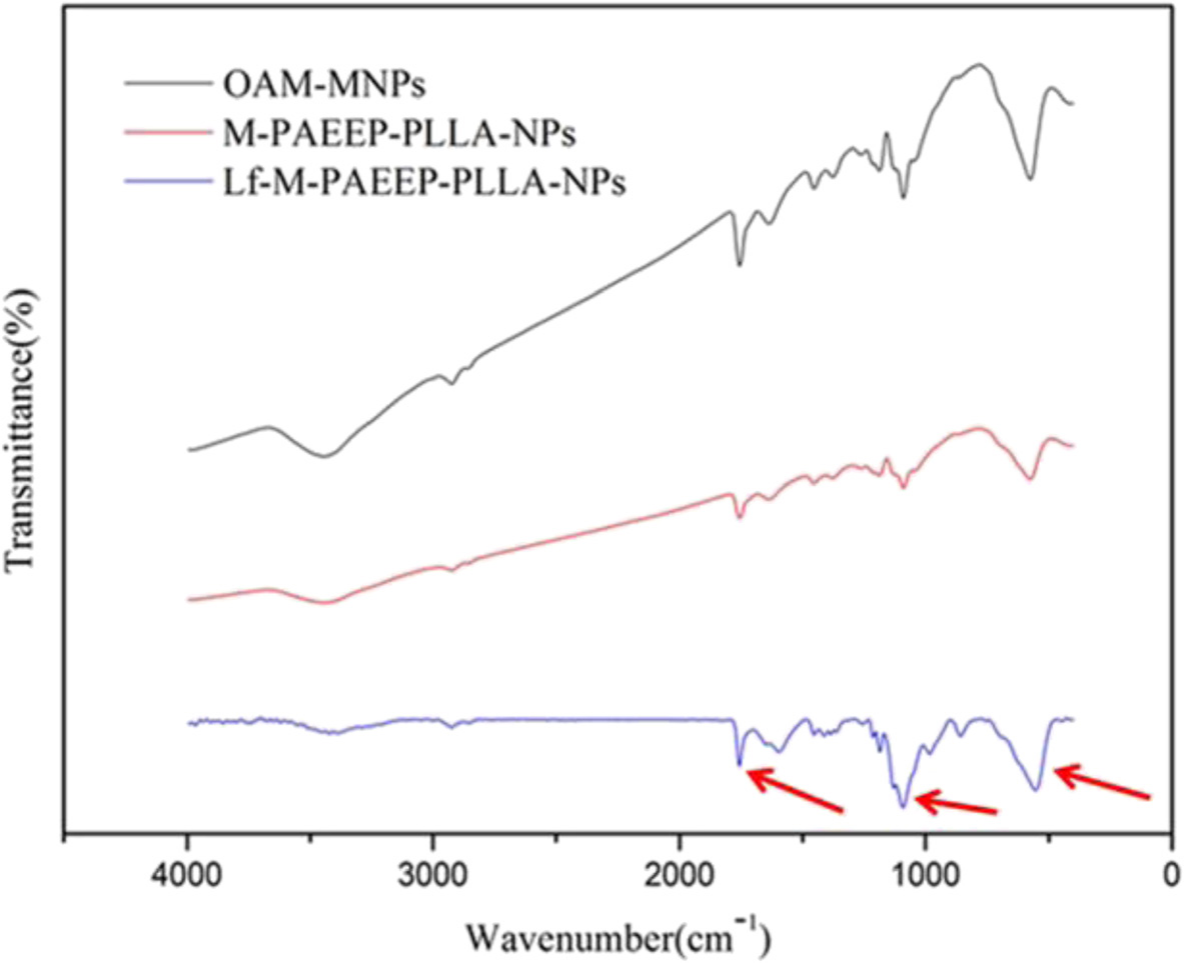
FTIR spectra of OAM-MNPs, M-PAEEP-PLLA-NPs, and Lf-M-PAEEP-PLLA-NPs

The FTIR spectra of OAM-MNPs, M-PAEEP-PLLA-NPs, and Lf-M-PAEEP-PLLA-NPs were shown in Fig. 3. All the nanoparticles have the typical absorption bands around 590 cm^−1^ refers to the stretch Fe–O vibration, which showed that iron oxide have been loaded in the copolymer nanoparticles. In the FTIR spectra of Lf-M-PAEEP-PLLA-NPs, the peak at 1600 cm^−1^ belongs to C=O stretch vibration and 1158 cm^−1^ to C-N stretch vibration, which indicated that Lf has conjugated to the copolymer nanoparticles.

**Fig. 4:**
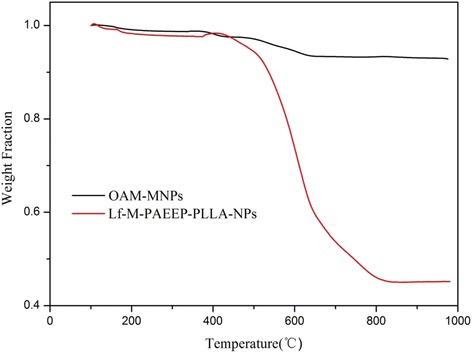
TGA curves of OAM-MNPs and Lf-M-PAEEP-PLLA-NPs

**Fig. 5:**
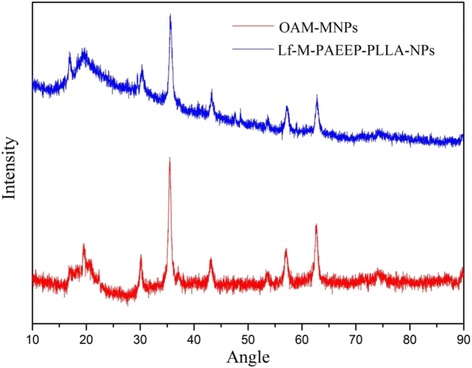
XRD curves of OAM-MNPs and Lf-M-PAEEP-PLLA-NPs

TGA of OAM-MNPs and Lf-M-PAEEP-PLLA-NPs are shown in Fig. 4. The content of iron oxide nanoparticles was 92.8 % in OAM-MNPs and 45.2 % in Lf-M-PAEEP-PLLA-NPs, respectively. In XRD analysis (Fig. 5), the positions and relative intensities of the diffraction peaks in OAM-MNPs and Lf-M-PAEEP-PLLA-NPs matched well with the standard XRD data of cubic Fe_3_O_4_ (JCPDS card No. 86–1359) [16].

**Fig. 6:**
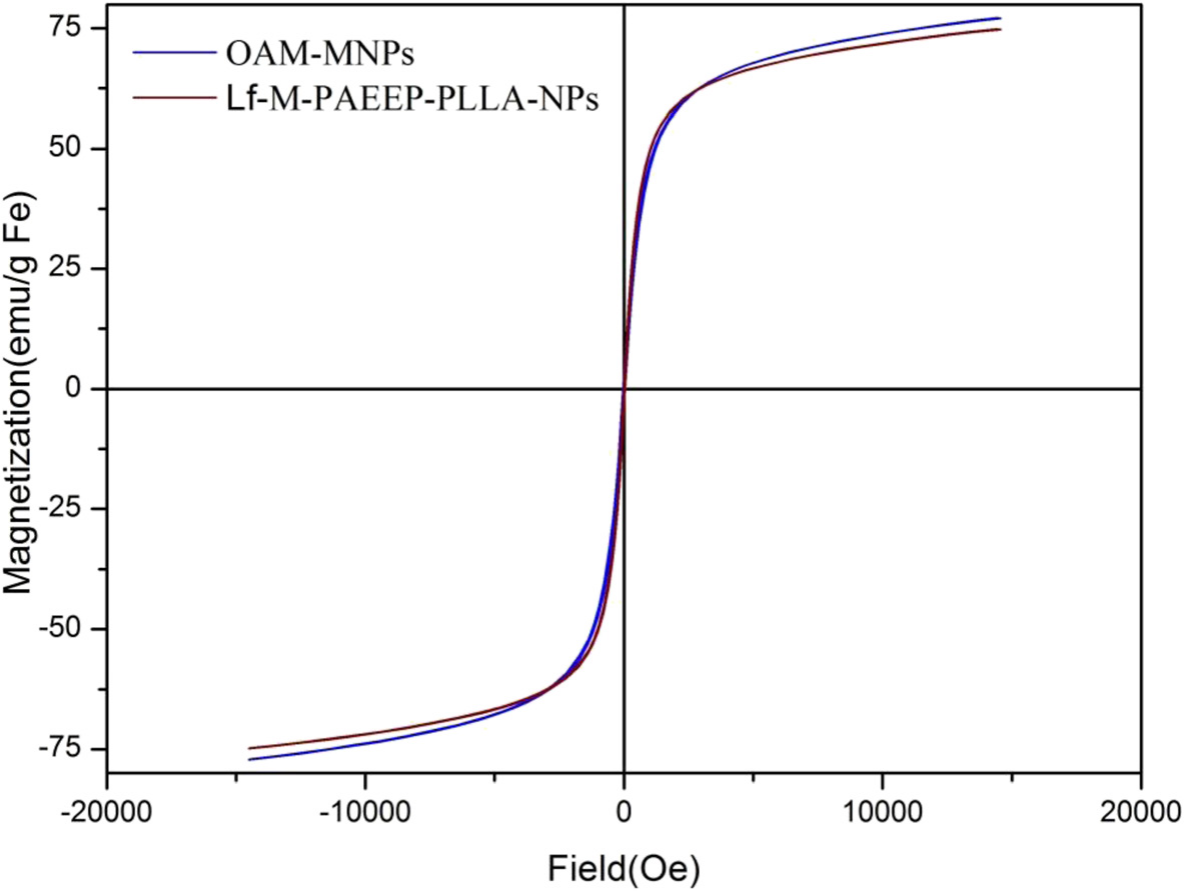
The magnetization curves of OAM-MNPs and Lf-M-PAEEP-PLLA-NPs

To evaluate the magnetic properties of Lf-M-PAEEP-PLLA-NPs, the nanoparticles were examined by a vibrating sample magnetometer. Figure 6 showed that both OAM-MNPs and Lf-M-PAEEP-PLLA-NPs exhibited good superparamagnetic behaviors (i.e., no hysteresis loop). The saturated magnetic intensity of OAM-MNPs and Lf-M-PAEEP-PLLA-NPs were 77.1 and 74.8 emu/g Fe, which indicated that the copolymer nanoparticles affected the magnetic properties of the iron oxide nanoparticles slightly.

### In Vitro Studies

The biocompatibility of M-PAEEP-PLLA-NPs and Lf-M-PAEEP-PLLA-NPs was evaluated by the cytotoxicity assay of C6 cells and HL-7702 cells. A viability of over 90 % in the two cell lines was maintained by increasing the M-PAEEP-PLLA-NPs and Lf-M-PAEEP-PLLA-NPs concentration from 12.5 to 100 μg/mL. The results indicated that M-PAEEP-PLLA-NPs and Lf-M-PAEEP-PLLA-NPs had no obvious cytotoxicity to C6 cells and HL-7702 cells within the concentration range used during the in vivo imaging. Thus, the cytotoxicity studies suggested that M-PAEEP-PLLA-NPs and Lf-M-PAEEP-PLLA-NPs had good biocompatibility and suitability for biomedical applications.

**Fig. 7:**
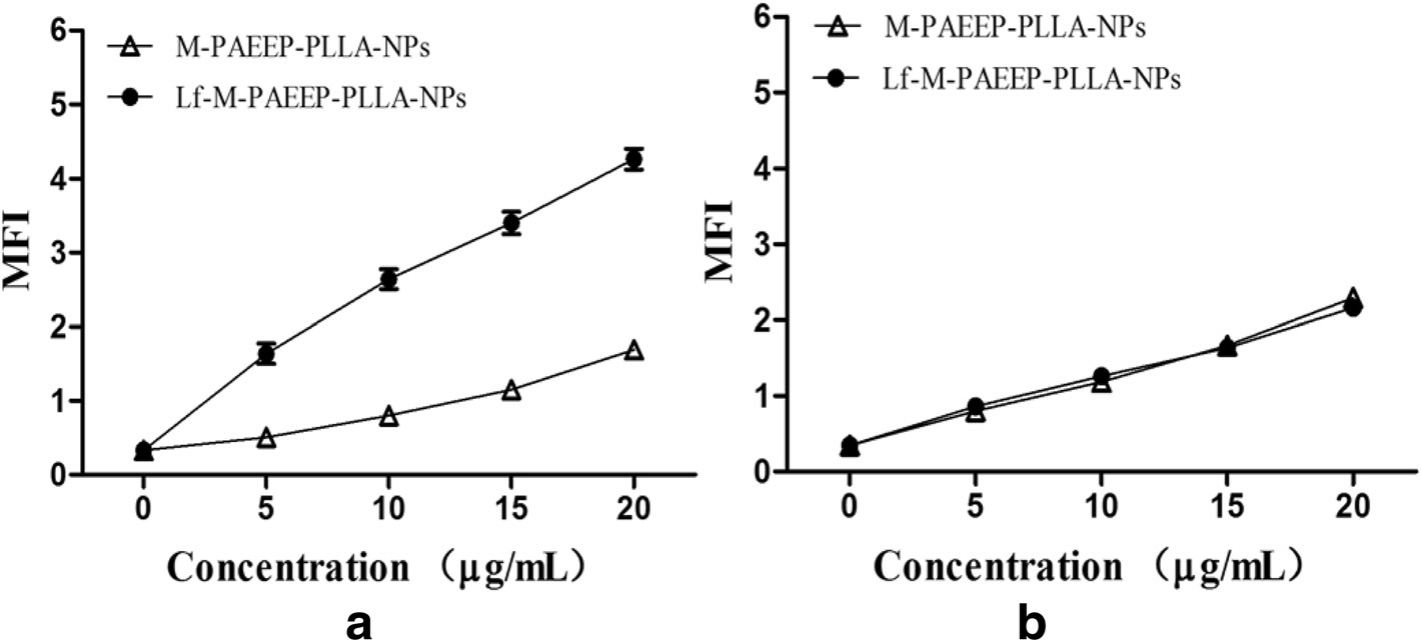
Effect of Lf on the cellular uptake. **a** Uptake curves of C6 cells. **b** Uptake curves of ECV304 cells. Data was reported as mean ± SD (*n* = 3)

C6 cells are rat glioma cells with highly low-density lipoprotein receptor-related protein (LRP1) expression. ECV304 cells are normal human umbilical vein endothelial cells (no LRP1 expression). Although LRP1 is expressed in some normal tissues, the level of LRP1 in tumor tissues is much higher. Lf is a LRP1 ligand, which is a promising targeting ligand for cancer cell. As shown in Fig. 7, for C6 cells, Lf-M-PAEEP-PLLA-NPs showed a significant increase in uptake compared with M-PAEEP-PLLA-NPs at the concentrations of 0~20 μg/mL. However, no obvious difference in uptake was observed in the ECV304 cells. The results clearly indicated that Lf-M-PAEEP-PLLA-NPs exhibited adequate active tumor cell targeting ability through the combination of the active targeting ligand of Lf.

**Fig. 8:**
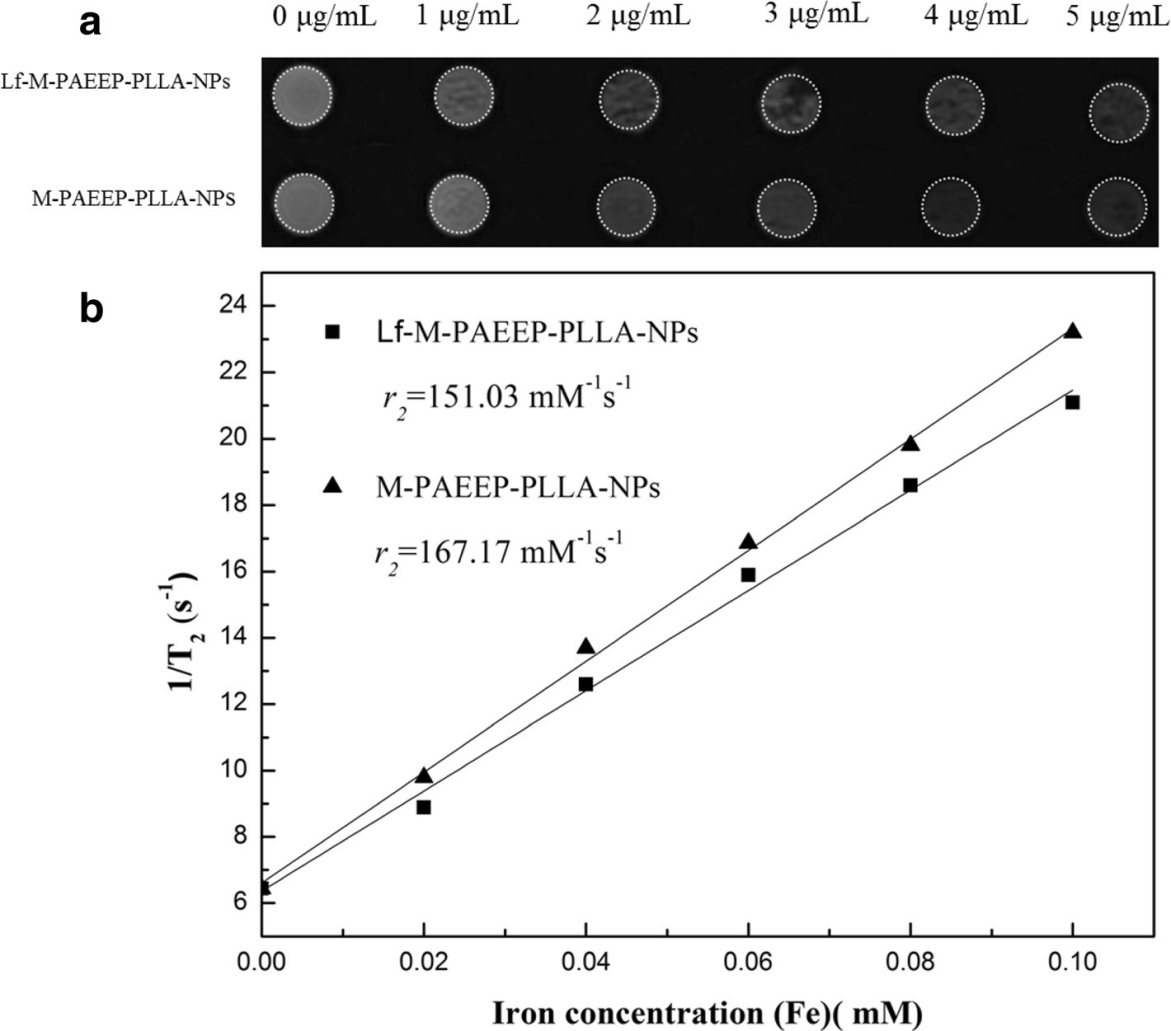
MR imaging abilities of the nanoparticles. **a** *T*_2_-weighted MR images of M-PAEEP-PLLA-NPs and Lf-M-PAEEP-PLLA-NPs. **b** Relaxivities (*r*_2_) of M-PAEEP-PLLA-NPs and Lf-M-PAEEP-PLLA-NPs measured at 300 K

The ability of M-PAEEP-PLLA-NPs and Lf-M-PAEEP-PLLA-NPs to enhance *T*_2_-weighted MR imaging was evaluated using 3.0-T MR scanner at 300 K. *T*_2_-weighted MR images for different iron concentrations of M-PAEEP-PLLA-NPs and Lf-M-PAEEP-PLLA-NPs suspension were acquired on the MRI instrument. As shown Fig. 8a, the signal intensities decayed significantly with the increase of the iron concentration. As shown Fig. 8b, a good linear correlation between *T*_2_ relaxation and the iron concentration was established as following [21, 22]:

$$ \raisebox{1ex}{$1$}\!\left/ \!\raisebox{-1ex}{${T}_2$}\right.=\raisebox{1ex}{$1$}\!\left/ \!\raisebox{-1ex}{${T}_2^0$}\right. + {r}_2\left[Fe\right] $$

$$ \raisebox{1ex}{$1$}\!\left/ \!\raisebox{-1ex}{${T}_2$}\right. $$ is the observed relaxation rate in the presence of magnetic nanoparticles, $$ \raisebox{1ex}{$1$}\!\left/ \!\raisebox{-1ex}{${T}_2^0$}\right. $$ is the relaxation rate of pure water, [*Fe*] is the concentration of the magnetic nanoparticles, and *r*_2_ is the transverse relaxivity. The relaxation rate of M-PAEEP-PLLA-NPs and Lf-M-PAEEP-PLLA-NPs were 167.2 and 151.3 mM^−1^s^−1^, respectively. However, the relaxation rate of SPIONs was usually 50~80 mM^−1^s^−1^ [7, 17]. Lf-M-PAEEP-PLLA-NPs could reduce the transverse relaxation time *T*_2_ more significantly compared with the SPIONs at the same concentration. The results might be due to the fact that Lf-M-PAEEP-PLLA-NPs made the SPIONs enriched in the polymer nanocarriers, which then made the number of SPIONs per volume higher than that of free hydrophilic SPIONs. The nanoparticles with higher *T*_2_ relaxation rate were suitable as a negative MRI contrast agents because of shortened relaxation time and decreased MRI signal. The results indicated that M-PAEEP-PLLA-NPs and Lf-M-PAEEP-PLLA-NPs encapsulated with OAM-MNPs could be used as MRI contrast agents.

### In Vivo MR Imaging

**Fig. 9:**
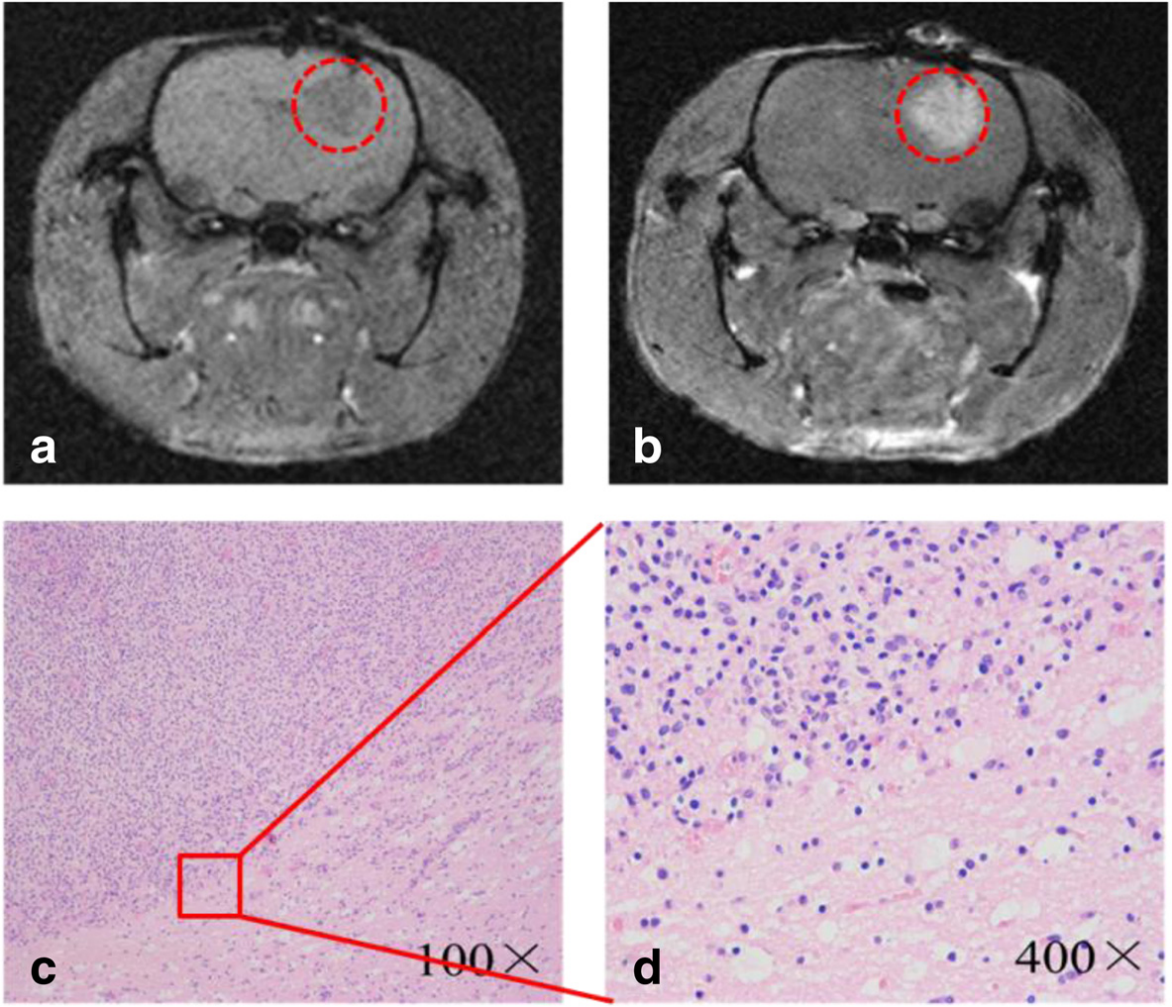
Characterization of the brain gliomas tumors. **a**, **b** MR images of gliomas before and post-injection of Gd-based contrast agent. **c**, **d** The results of H&E staining

Figure 9a showed the image of a high grade glioma tumor 14 days post-inoculation of C6 cells. Figure 9b showed the enhancement on *T*_1_-weighted imaging after administration of Gd-based contrast agent. The H&E staining of tumor tissues revealed higher density of tumor cells than normal cells. The boundaries of tumor cells were unclear, indicating the infiltration into the surrounding normal brain tissue. We also observed pronounced nuclear and cytoplasmic polymorphism, intratumoral necrosis, and mitotic figures (Fig. 9c, d). The results confirmed the successful establishment of rat glioma model.

**Fig. 10:**
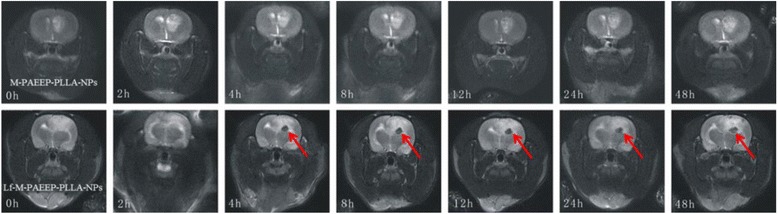
*T*_2_-weighted MR images of gliomas before and post-injection of Lf-M-PAEEP-PLLA-NPs

Figure 10 was the *T*_2_-weighted MRI of brain glioma before and after injection of M-PAEEP-PLLA-NPs and Lf-M-PAEEP-PLLA-NPs. We observed significantly decreased signal in tumor region 4 h after injection of Lf-M-PAEEP-PLLA-NPs, and the area of the decreased *T*_2_-weighted signal increased and even lasts 48 h. However, we did not observe such changes in signal with the injection of M-PAEEP-PLLA-NPs. The results indicated that Lf-M-PAEEP-PLLA-NPs exhibited the contrast-enhanced MRI and lower signal intensity compared to M-PAEEP-PLLA-NPs.

**Fig. 11:**
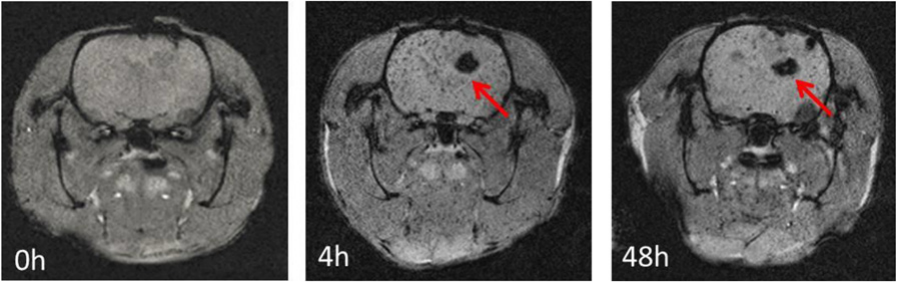
SWI MR images of gliomas before and post-injection of Lf-M-PAEEP-PLLA-NPs

SWI is based on high-resolution, three-dimensional, fully velocity-compensated gradient-echo sequences using both magnitude and phase images [23]. It could be used as a complementary source of information in the detection of mineral deposition, such as iron and copper [24]. These highly paramagnetic materials with larger magnetic susceptibility could cause local magnetic field changes and protons dephasing, thus leading to low signal on the phase diagram. Figure 11 showed SWI images before and after injection of Lf-M-PAEEP-PLLA-NPs. The MR hypointensity of the tumors 4 and 48 h after injection were significantly higher than that before injection. These hypointense regions indicated the presence of iron oxide nanoparticles within the tumor tissue. In the study, no significant MRI signal changes were observed in tumor tissue with injection of M-PAEEP-PLLA-NPs.

**Fig. 12:**
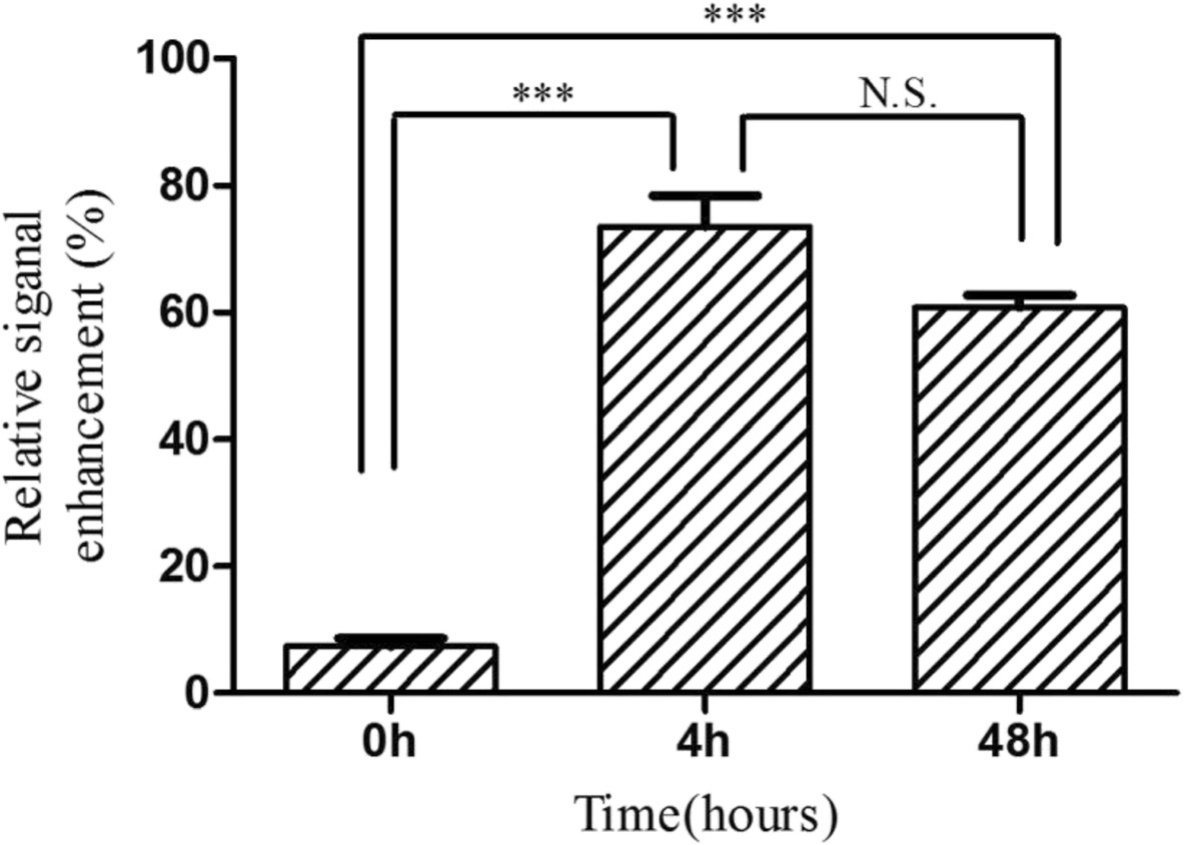
The relative signal enhancement of the brain tumor in the *T*_2_ MR images

**Fig. 13:**
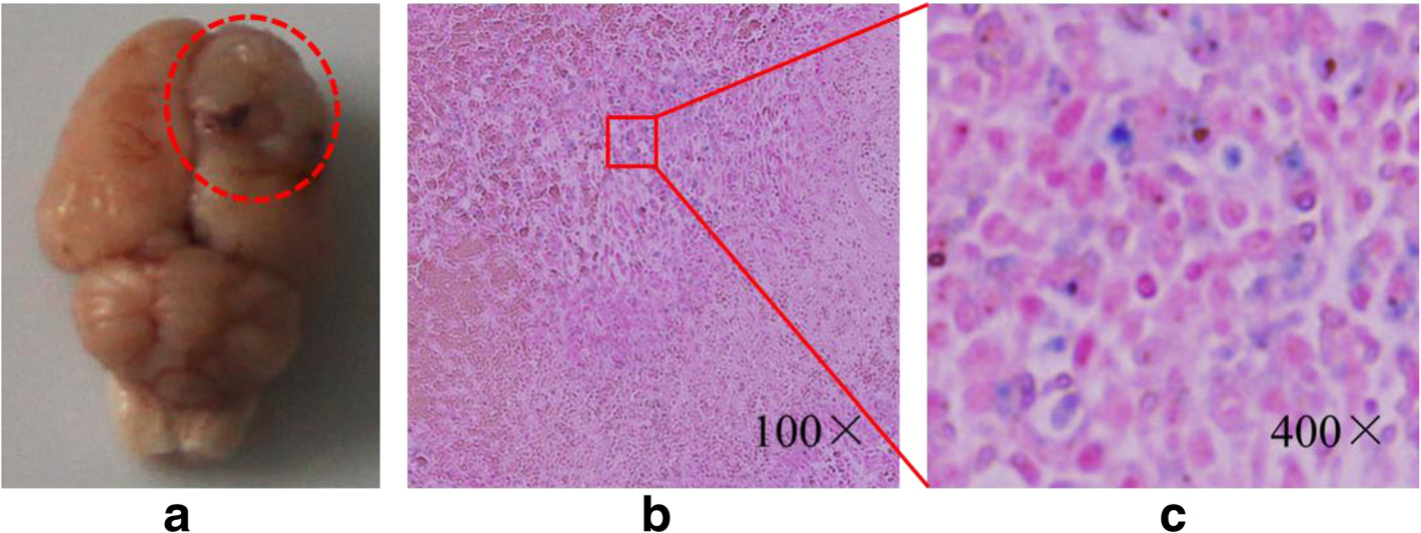
The appearance (**a**) and histological sections of glioma with Prussian blue staining (**b**, **c**)

Figure 12 showed the RSE of Lf-M-PAEEP-PLLA-NPs, which were calculated according to the SI of the defined tumor area and the back muscle adjacent to the tumor. The RSE of Lf-M-PAEEP-PLLA-NPs after injection 0, 4, and 48 h were 6.5, 78.2, and 62.5 %, respectively. The histological sections of glioma with Prussian blue staining were shown in Fig. 13. Significant amount of iron oxide nanoparticles was still clearly observed around the vascular region of the tumor tissue slices at 48 h of injection. At high magnification (400×), Lf-M-PAEEP-PLLA-NPs were observed to be located intracellularly. The results indicated that the enhanced MR *T*_2_-weighted images of brain glioma in Figs. 10 and 11 were due to the presence of Lf-M-PAEEP-PLLA-NPs. Consistently, poor in vivo uptake of M-PAEEP-PLLA-NPs by glioma cells was also verified using the same staining method as a comparison.

The results indicated strong, long-lasting, tumor-targeting, and contrast-enhanced MRI ability of Lf-M-PAEEP-PLLA-NPs owing to the selectively accumulation in brain glioma tissue. Moreover, there were studies that showed that Lf mediates the blood–brain barrier (BBB) transportation by the reorganization of Lf receptor (LfR) on the BBB [25, 26]. With the unidirectional receptor-mediated transcytosis, polymer nanocarriers conjugated with Lf could cross the BBB and enter into the brain. So we can conclude that Lf could strengthen the ability of the Lf-M-PAEEP-PLLA-NPs through the BBB and binding to brain glioma cells with LfR. These suggested that Lf-M-PAEEP-PLLA-NPs could be an optimal contrast agent for brain glioma targeting MR imaging.

## Conclusions

In this study, oleylamine-coated superparamagnetic Fe_3_O_4_ nanoparticles were successfully synthesized and encapsulated in PAEEP-PLLA nanoparticles with ultrasonic emulsification and solvent evaporation method. Lf, an effective targeting ligand for brain glioma cells, was conjugated to the Lf-M-PAEEP-PLLA-NPs. We observed strong, long-lasting, tumor-targeting, and contrast-enhanced MR imaging ability of Lf-M-PAEEP-PLLA-NPs by in vitro and in vivo MRI studies. Our results suggest that Lf-M-PAEEP-PLLA-NPs represent a promising nano-sized MRI contrast agent for brain glioma targeting.

### Consent

The authors have given their consent for the case reports to be published. Written informed consent was obtained from the author for publication of this case report and any accompanying images. A copy of the written consent is available for review by the Editor-in-Chief of this journal.

## Abbreviations

C6: rat C6 glioma cell
FTIR: Fourier transform infrared
Gd: gadolinium
H&E: hematoxylin and eosin
HL7702: human normal liver cell
Lf: lactoferrin
MRI: magnetic resonance imaging
OAM-MNPs: oleylamine-coated Fe_3_O_4_ magnetic nanoparticles
PAEEP-PLLA: poly(aminoethyl ethylene phosphate)/poly(L-lactide)
PEG: polyethylene glycol
SWI: susceptibility weighted imaging.
TEM: transmission electron microscopy
TGA: thermogravimetric analysis
XRD: X-ray diffraction
